# Identification and Biological Evaluation of a Novel CLK4 Inhibitor Targeting Alternative Splicing in Pancreatic Cancer Using Structure‐Based Virtual Screening

**DOI:** 10.1002/advs.202416323

**Published:** 2025-03-24

**Authors:** Chun‐Lin Yang, Yi‐Wen Wu, Huang‐Ju Tu, Yun‐Hsuan Yeh, Tony Eight Lin, Tzu‐Ying Sung, Mu‐Chun Li, Shih‐Chung Yen, Jui‐Hua Hsieh, Ming‐Chin Yu, Sen‐Yung Hsieh, Hsing‐Pang Hsieh, Shiow‐Lin Pan, Kai‐Cheng Hsu

**Affiliations:** ^1^ Graduate Institute of Cancer Biology and Drug Discovery College of Medical Science and Technology Taipei Medical University Taipei 110301 Taiwan; ^2^ Ph.D. Program for Cancer Molecular Biology and Drug Discovery College of Medical Science and Technology Taipei Medical University Taipei 110301 Taiwan; ^3^ Institute of Biotechnology and Pharmaceutical Research National Health Research Institutes Miaoli County 350401 Taiwan; ^4^ Biomedical Translation Research Center Academia Sinica Taipei 115202 Taiwan; ^5^ Warshel Institute for Computational Biology School of Medicine The Chinese University of Hong Kong (Shenzhen) Shenzhen Guangdong 518172 China; ^6^ Division of Translational Toxicology National Institute of Environmental Health Sciences National Institutes of Health Durham NC 27709 USA; ^7^ College of Medicine Chang Gung University Taoyuan 333323 Taiwan; ^8^ Department of Surgery New Taipei Municipal TuCheng Hospital (Built and Operated by Chang Gung Medical Foundation) Tucheng New Taipei City 236043 Taiwan; ^9^ Graduate Institute of Clinical Medical Sciences Chang Gung University Guishan Taoyuan 333323 Taiwan; ^10^ Department of Gastroenterology and Hepatology Chang Gung Memorial Hospital Linkou Taoyuan 333423 Taiwan; ^11^ Department of Chemistry National Tsing Hua University Hsinchu 300044 Taiwan; ^12^ TMU Research Center of Cancer Translational Medicine Taipei Medical University Taipei 110301 Taiwan; ^13^ Cancer Center Wan Fang Hospital, Taipei Medical University Taipei 116079 Taiwan

**Keywords:** alternative splicing, CLK4, kinase inhibitor, pancreatic cancer, structure‐based virtual screening

## Abstract

Pancreatic cancer is an aggressive malignancy with a poor prognosis and limited treatment options. Cdc‐like kinase 4 (CLK4), a kinase that regulates alternative splicing by phosphorylating spliceosome components, is implicated in aberrant splicing events driving pancreatic cancer progression. In this study, we established a computational model that integrates pharmacological interactions of CLK4 inhibitors with an improved hit rate. Through this model, we identified a novel CLK4 inhibitor, compound 150441, with a 50% inhibitory concentration (IC_50_) value of 21.4 nm. Structure‐activity relationship analysis was performed to investigate key interactions and functional groups. Kinase profiling revealed that compound 150441 is selective for CLK4. Subsequent in vitro assays demonstrated that this inhibitor effectively suppressed cell growth and viability of pancreatic cancer cells. In addition, it inhibited the phosphorylation of key splicing factors, including serine‐ and arginine‐rich splicing factor (SRSF) 4 and SRSF6. Cell cycle analysis further indicated that the compound induced G2/M arrest, leading to apoptosis. RNA‐seq analysis revealed that the compound induced significant changes in alternative splicing and key biological pathways, including RNA processing, DNA replication, DNA damage, and mitosis. These findings suggest that compound 150441 has promising potential for further development as a novel pancreatic cancer treatment.

## Graphical abstract

Pancreatic cancer is a highly aggressive malignancy with limited treatment options. CLK4 regulates alternative splicing, contributing to cancer progression. This study establishes a computational model to identify CLK4 inhibitors, leading to compound 150441 (IC_50_: 21.4 nm). It selectively inhibits CLK4, suppresses cancer cell growth, induces G2/M arrest, and alters RNA splicing, highlighting its therapeutic potential.

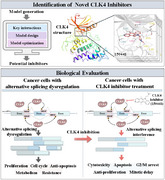

## 1 Introduction

Pancreatic cancer remains one of the most aggressive and high‐risk malignancies to date. It is associated with extremely poor prognostic outcomes and is characterized by a notably low survival rate.^[^ ^1^, ^2^ ^]^ Early‐stage pancreatic cancer is often asymptomatic, and the disease typically only becomes apparent after the tumor invades surrounding tissues or metastasizes to distant organs. As a result, the 5‐year survival rate for pancreatic cancer is only 2–9%.^[^ ^3^, ^4^ ^]^ Even with treatment, within 24 months postsurgery, 60% of patients develop distant metastases, significantly contributing to mortality.^[^ ^1^, ^5^ ^]^ In addition to surgical tumor resection, current treatment guidelines incorporate chemotherapy. Although chemotherapy can improve survival rates in patients with pancreatic cancer, traditional chemotherapeutic agents currently in use have several limitations. The most concerning limitation is their limited efficacy, as the median overall survival of patients receiving conventional chemotherapy does not exceed 2 years.^[^ ^1^ ^]^ Additionally, traditional chemotherapeutic agents not only target cancer cells but also affect normal cells, leading to unexpected side effects. Therefore, it is necessary and urgent to develop targeted therapies that can improve clinical outcomes by inhibiting specific targets in pancreatic cancer cells and potentially have fewer side effects.

RNA splicing is a crucial biological process where introns are removed from precursor messenger RNAs (pre‐mRNAs), resulting in mature RNAs composed of exons.^[^ ^6^, ^7^ ^]^ Among various splicing types, alternative splicing (AS) is a key player in cancer development. Studies reveal that many cancers exhibit aberrant AS compared to normal tissues.^[^ ^8^, ^9^ ^]^ Aberrant AS can significantly impact various hallmarks of cancer, including cell proliferation, apoptosis, epithelial‐mesenchymal transition (EMT), and tumor immune evasion.^[^ ^2^ ^]^ Studies reveal that AS is regulated by splicing factors (SFs) through interactions with pre‐mRNA molecules.^[^ ^6^, ^7^, ^10^ ^]^ Notably, some SFs are associated with pancreatic cancer, such as serine‐ and arginine‐rich (SR) splicing factor 1 (SRSF1), SRSF5, and SRSF6.^[^ ^1^, ^2^ ^]^ A study has shown that SRSF1 is upregulated in pancreatic cancer cells, and knocking out SRSF1 inhibits cell migration, invasion, and EMT.^[^ ^11^ ^]^ Phosphorylation of SRSF5 regulates AS events in pancreatic cancer cells.^[^ ^12^ ^]^ In addition, the activation of SRSF6 also contributes to the metastasis of pancreatic cancer.^[^ ^5^ ^]^ These findings indicate the crucial roles of AS and SFs in pancreatic cancer.

CLK4, or Cdc2‐like kinase 4, is a dual‐specificity kinase belonging to the LAMMER kinase family.^[^ ^5^, ^8^ ^]^ CLK4 regulates the AS of pre‐mRNA by phosphorylating serine‐ and arginine‐rich splicing proteins within the spliceosome.^[^ ^13^, ^14^ ^]^ Therefore, the aberrant AS in several cancers can be moderated by inhibiting CLK4 activity. Moreover, higher CLK4 expression is associated with poorer patient survival in triple‐negative breast cancer (TNBC). Silencing CLK4 in an aggressive mesenchymal‐like subtype (MES) of TNBC reduces the expression of genes involved in metastasis and lessens the invasive behavior of these cancer cells.^[^ ^15^ ^]^ Therefore, CLK4 represents a promising therapeutic target for development. Currently, several CLK4 inhibitors have been developed, including a few that have entered phase I clinical trials. For example, SM08502 (Cirtuvivint), which inhibits CLK4, showed efficacy across 17 colorectal cancer (CRC) cell lines and inhibited the proliferation of six human gastric cancer cell lines with various mutations.^[^ ^16^ ^]^ Furthermore, it exhibited significant tumor growth inhibition in xenograft models of CRC and gastric cancer.^[^ ^16^ ^]^ In addition, TG003, a discovery‐stage compound, showed anticancer activity in prostate cancer cells and altered splicing of cancer‐related genes.^[^ ^17^ ^]^ However, existing drugs often exhibit off‐target activity, which can increase the risk of adverse effects. Therefore, there is a need to develop a new CLK4 inhibitor with high selectivity for cancer treatment through targeting the aberrant AS.

**Figure 1:**
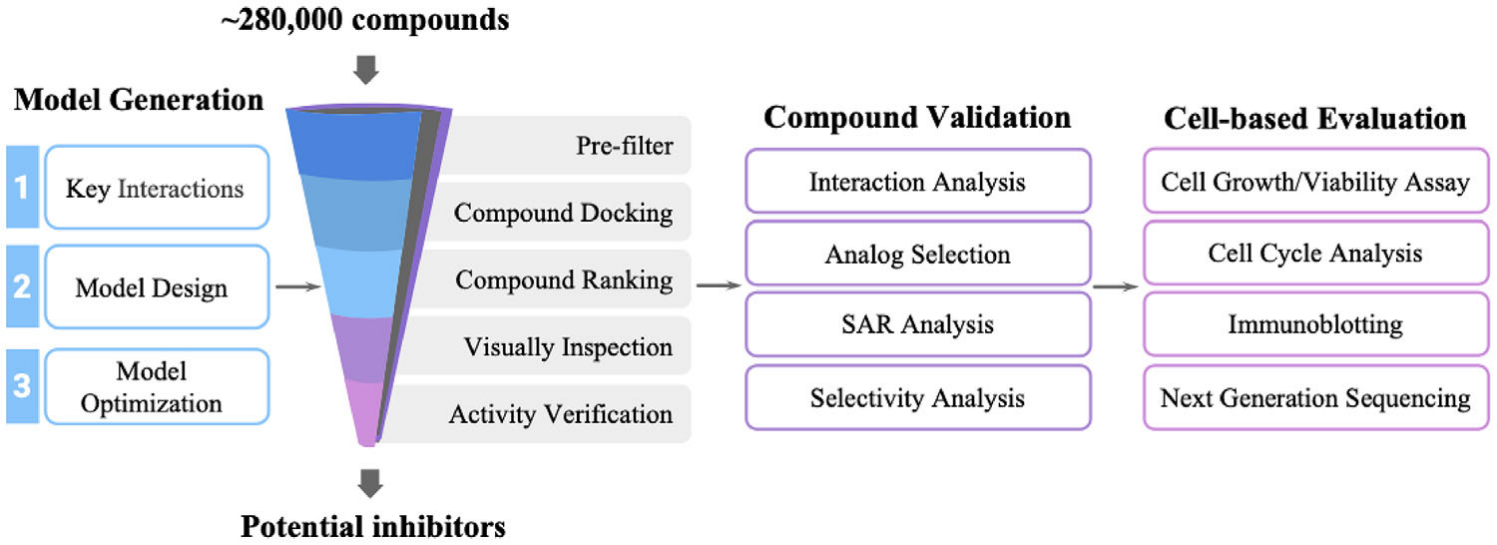
Workflow of the study. Initially, a computational model for identifying CLK4 inhibitors was first developed. Then, the model was applied to identify potential inhibitors. Selected compounds were validated using enzymatic assays. The effects of identified inhibitors on cancer cells were further evaluated.

In this study, we performed structure‐based virtual screening (SBVS) to identify novel CLK4 inhibitors, followed by experimental validation of the identified compounds. The workflow of this study is shown in **Figure** **1**. First, we identified the pharmacological interactions of CLK4 and established a computational model. Pharmacological interactions can be applied to improve the hit rate in SBVS.^[^ ^14^, ^18^ ^]^ Subsequently, compounds from the National Cancer Institute (NCI) library were screened, and potential inhibitors were selected and tested for their inhibitory activity by enzymatic assays. The compound with the highest potency was selected for further exploration, including a search for its analogs and a structure‐activity relationship (SAR) analysis of these analogs. Validated inhibitors were then evaluated for their effects on the viability and proliferation of pancreatic cancer cells. The compound with the best potency was further assessed for its ability to inhibit the phosphorylation of downstream proteins (i.e., SRSFs) and its impact on the cell cycle. We also tested the compound for selectivity against a panel of kinases from various families. Finally, an RNA sequencing (RNA‐seq) analysis was conducted to examine the global AS changes induced by the compound. In summary, this study identified a novel inhibitor that serves as a crucial starting point for developing potent CLK4 inhibitors for treating pancreatic cancer.

## 2 Result

### 2.1 Establishment of Pharmacological Model

We first evaluated the docking procedure by performing a redocking analysis. Currently, only one structure of CLK4 (PDB ID: 6FYV) is available, with a resolution of 2.46 Å and no missing residues in the binding site. As such, it was selected for docking and screening analysis. The redocking procedure demonstrated that the predicted docking pose is similar to the conformation of the co‐crystallized ligand (Figure S1, Supporting Information). These results suggest that 6FYV and the docking program are suitable for virtual screening.

**Figure 2:**
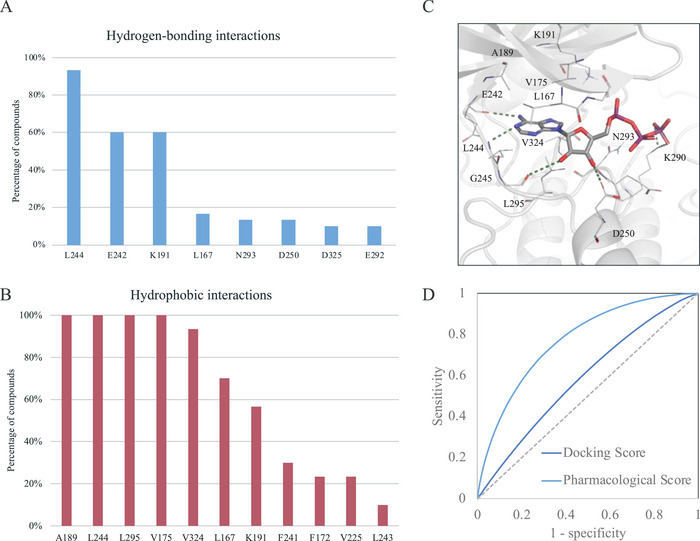
Analysis of pharmacological interactions for CLK4. The analysis includes two types of interactions: A) hydrogen‐bonding and B) hydrophobic interactions. Interactions occurring with a frequency of 50% or higher are considered pharmacological interactions. C) Docking pose of ATP. Hydrogen bonds are shown as green dashes. Binding site residues are depicted as lines and labeled as shown. D) Comparison of scoring functions using docking score alone and pharmacological interaction score.

To increase the hit rate of structure‐based virtual screening, we next identified pharmacological interactions between inhibitors and the CLK4 binding site. Inhibitors with a 50% inhibitory concentraton (IC_50_) value of ≤ 1 µm were collected from ChEMBL. From this set, 60 inhibitors with diverse structures were selected and docked into the binding site. The top 30 inhibitors were then selected based on their docking score and were used to determine pharmacological interactions (**Figure** **2**). An interaction involving at least 50% of the 30 inhibitors was considered a pharmacological interaction. Three pharmacological hydrogen‐bonding interactions were identified, including the interacting residues L244, E242, and K191 (Figure 2A). Notably, the first two residues, L244 and E242, were located in the hinge region and formed hydrogen bonds with the adenosine group of ATP (Figure 2C). The hydrogen‐bonding interaction with residue K191 exhibited a 60% frequency among the diverse inhibitors, which was not observed in ATP. The analysis also identified seven pharmacological hydrophobic interactions involving the residues A189, L244, V175, L167, L295, V324, and K191 (Figure 2B). Notably, the first four residues formed a pocket that accommodates the adenosine of ATP, while residues L167 and L295 make contact with the ribose group of ATP (Figure 2C). These pharmacological interactions play a critical role in the inhibitory action against CLK4.

Next, we evaluated the performance of the pharmacological model in identifying inhibitors. Thirty known CLK4 inhibitors and 990 compounds randomly selected from the Available Chemical Directory (ACD)^[^ ^19^ ^]^ were docked into CLK4. These compounds were ranked based on either their docking score alone or their pharmacological interaction score. For each compound, the pharmacological score, *S*(*i*), was calculated as follows:

(1) $$S\left(i\right)=N\left(i\right)+\left(-0.01\right)D\left(i\right)$$

where *N*(*i*) represents the number of pharmacological interactions compound *i* forms, and *D*(*i*) is the docking score of compound *i*. The receiver operating characteristic (ROC) curve analysis showed that pharmacological scoring yielded a better high area under the curve (AUC) value compared with the docking scoring, indicating that the pharmacological scoring provided better screening performance (Figure 2D).

We further optimized the hit rate of the pharmacological model by incorporating the analysis of inactive compounds. An additional 30 inactive CLK4 compounds with an IC_50_ value of >10 µm were selected and docked into the binding site. A comparative interaction analysis between the inactive and active compounds was performed to identify interactions with varying frequencies. The analysis revealed two crucial hydrophobic interactions formed with the residue L244 and L167, which showed the most significant differences (Figure S2A, Supporting Information). Subsequently, these two interactions were integrated into the pharmacological model to evaluate their potential to improve the model performance. In addition, different weights were applied to these interactions and tested. For each compound, the optimized pharmacological score, *OS*(*i*), was calculated using three different equations:

(2) $$\mathrm{OS}\left(i\right)=S\;\left(i\right)+1\left(L167\left(i\right)+L244\left(i\right)\right)$$

(3) $$\mathrm{OS}\left(i\right)=S\;\left(i\right)+2\left(L167\left(i\right)+L244\left(i\right)\right)$$

(4) $$\mathrm{OS}\left(i\right)=S\;\left(i\right)+3\left(L167\left(i\right)+L244\left(i\right)\right)$$

where *S*(*i*) represents the pharmacological score of compound *i*, *L167*(*i*) is 1 if the compound forms a hydrophobic interaction with residue L167; otherwise, *L167*(*i*) is 0, and *L244*(*i*) is 1 if the compound forms a hydrophobic interaction with residue L244; otherwise, *L244*(*i*) is 0. The compounds were ranked based on their optimized pharmacological scores, and the performance of the three equations was evaluated (Figure S2B, Supporting Information). The results indicated that Equation (4) demonstrated the best performance, with an AUC of the ROC curve of 0.81. In contrast, the AUC‐ROC was 0.77 when using the pharmacological score alone. Therefore, Equation (4) was selected for further identification of potential CLK4 inhibitors.

### 2.2 Identification and Validation of Potential Inhibitors

Next, we applied the optimized pharmacological model to screen compounds from the NCI library, which consists of ≈280,000 compounds. Compounds that contain the PAINS structures, have quantitative estimate of drug‐likeness (QED) scores below 0.25 or violate “Lipinski's and Veber's Rules” were excluded. The remaining compounds were docked into the binding site of CLK4 and ranked according to their pharmacological scores, calculated using Equation (4). The top 400 compounds were clustered by their structure similarity. Representative compounds from each cluster were selected and visually inspected. Based on their availability and visual inspection, 15 compounds were ultimately selected for testing. The selected compounds were then evaluated for inhibitory activity at a concentration of 10 µm using LanthaScreen Binding technology. The results revealed that compound 150441 exhibited the highest inhibitory effect on CLK4 activity, with an inhibition rate of 98% (Table S1, Supporting Information). These findings suggest that pharmacological interactions are useful for discovering CLK4 inhibitors and were successfully applied to identify a novel CLK4 inhibitor.

### 2.3 Interaction Analysis

**Figure 3:**
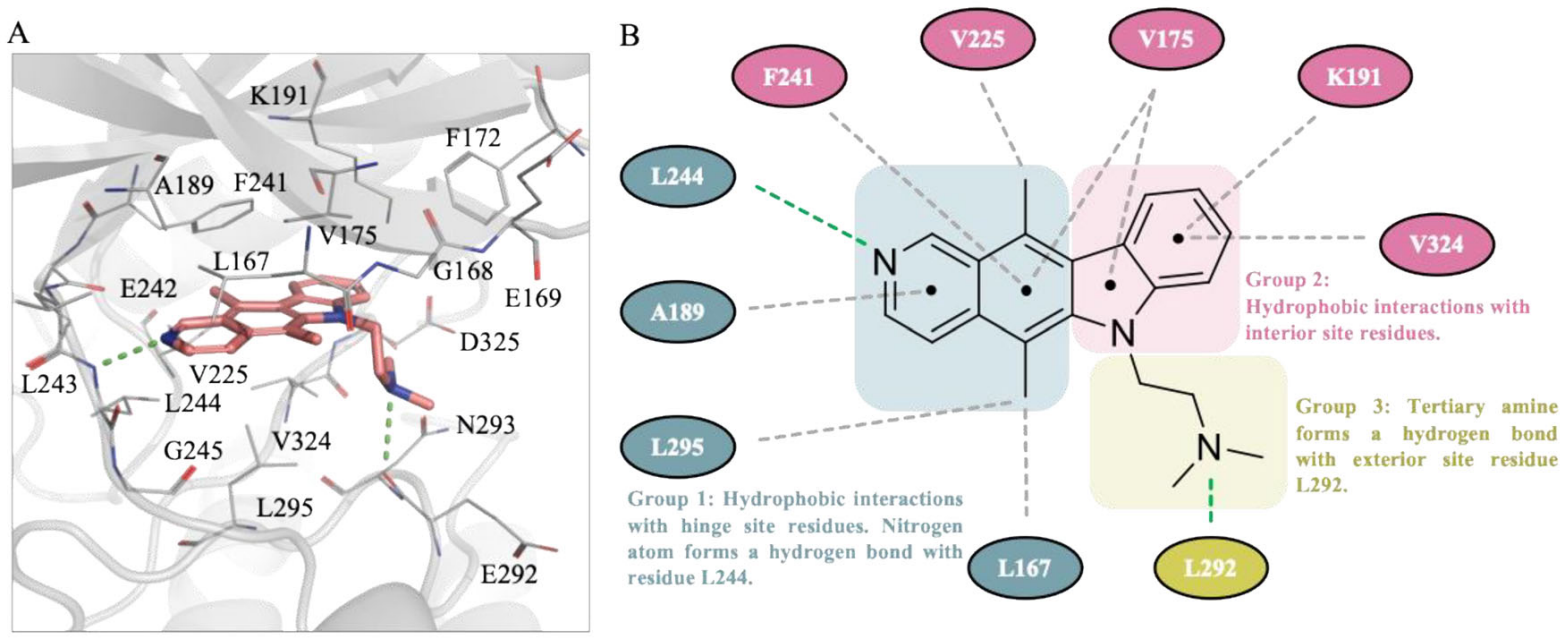
Docking pose and interactions of compound 150 441 in the CLK4 binding site. A) Compound 150441 (salmon) shows favorable occupation of the CLK4‐binding site (gray). Hydrogen bonds are represented as green dash lines. Binding site residues are listed as shown. B) The 2D interaction figure shows hinge residues (blue) interact with isoquinoline, interior residues (pink) interact with indole, and exterior residues (lime) interact with dimethylethylamine. Hydrogen bonds are represented as green dash lines, and hydrophobic interactions are represented as gray dot lines.

We performed an interaction analysis to study the molecular interactions of compound 150441. The docking results showed that compound 150441 occupied the binding site of CLK4 (**Figure** **3**A; Figure S3, Supporting Information). The main scaffold of compound 150441 is a heterotetracyclic structure that includes an isoquinoline (Group 1) and an indole ring (Group 2), along with a dimethylethylamine side chain (Group 3) (Figure 3B). Group 1 forms a hydrogen bond with residue L244. The docking pose of ATP shows that the adenine scaffold of ATP occupies a position similar to Group 1 and forms the same hydrogen bonds with L244 (Figure 2C). Moreover, Group 1 is sandwiched by various residues, yielding hydrophobic interactions with L167, V175, A189, V225, F241, L244, and L295 (Figure 3A). The primary interactions established by Group 2 are hydrophobic, involving the side chains of residues V175, K191, and V324. Group 3 forms a hydrogen bond with residue E292 (Figure 3B). Together, these interactions suggest that compound 150441 can bind to the CLK4 binding site and further interfere with the activity of CLK4.

### 2.4 Structure‐Activity Relationship Analysis

**Figure 4:**
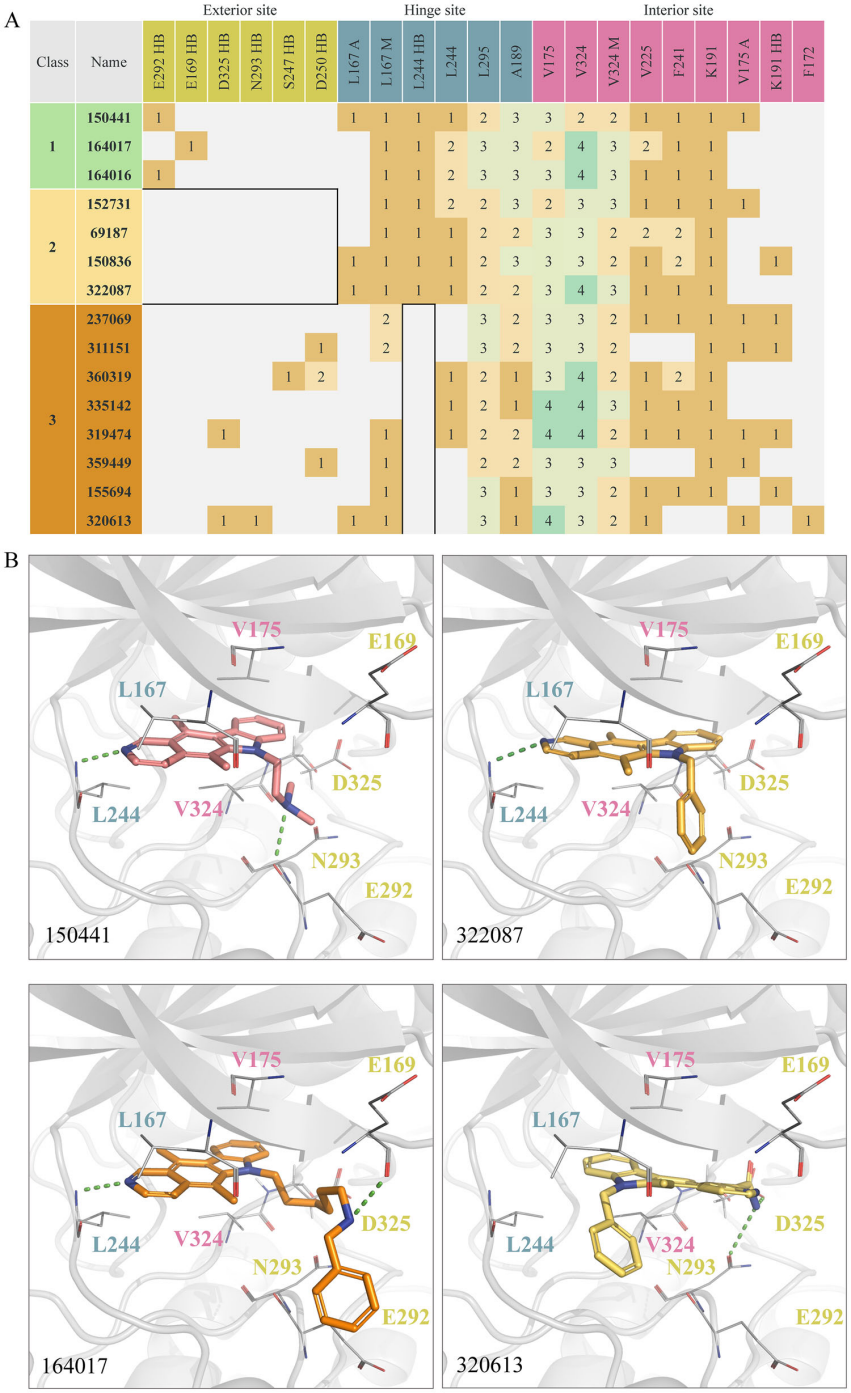
SAR analysis of 150441 and its analogs. A) Interaction profile of the compounds. The compounds were categorized into three classes based on their inhibitory activity. The most potent class is shown as green, and the weakest class is shown as orange. Residues in the CLK4 binding pocket are categorized into exterior, hinge, and interior sites. HB, A, and M stand for hydrogen‐bonding interaction, alkyl hydrophobic interaction, and mixed hydrophobic interaction, respectively. B) Docking poses of the compounds. Hydrogen bonds are shown as green dashes. Exterior, hinge, and interior residues are shown in lime, blue, and pink, respectively.

To better understand the binding interactions of compound 150441, a structure‐activity relationship (SAR) analysis was conducted. Fifteen analogs were selected from the NCI library and tested for their inhibitory activity (**Table** **1**). Compounds showing over 50% inhibitory activity were further evaluated for their IC_50_ values (**Table** **2**; Figure S4, Supporting Information). The analogs were categorized into three classes based on their interactions and inhibitory activities (**Figure** **4**). Class 1 includes compounds 150441, 164016, and 164017. The compounds in this class form hydrogen bonds with residues E292 or E169 at the exterior site. Compounds 150441 and 164016 both contain an ethylamine functional group, allowing them to form hydrogen bonds with residue E292. Compound 164017 has a hydrogen‐bonding interaction with residue E169. In addition, compound 150 441 yields hydrophobic alkyl interactions with residues L167 and V175, which may contribute to its slightly better inhibitory activity compared to the other compounds.

**Table 1:** Inhibitory activity of 150441 analogs.

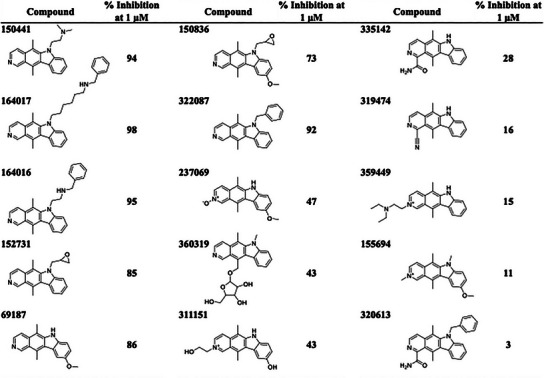

**Table 2:** IC_50_ values of identified inhibitors.

Compound	IC_50_ [nm]
150441	21.4 nm
164017	28.6 nm
164016	34.8 nm
152731	38.2 nm
69187	98.8 nm
150836	101.1 nm
322087	120.6 nm

Compounds in Class 2 lack the hydrogen bond with exterior residues E292 or E169, leading to lower activities compared to those in Class 1 (Figure 4). The main difference arises from the absence of an alkylamine moiety in Class 2 compounds. For example, compound 152731, lacking the ethylamine group compared to compound 150441, cannot form a hydrogen bond with residue E292. Compounds 69187 and 150836 not only lack the hydrogen bonds with exterior residues but also the mixed hydrophobic interaction with residue V324. Compound 322087 lacks a hydrophilic moiety in Group 3 and has fewer interactions with residues A189 and F241, resulting in the lowest inhibitory activity in Class 2. Furthermore, compound 150836 differs from compound 152731 by the presence of a methoxy group, which reduces its inhibitory activity by limiting interactions with the hinge residue L244.

Class 3 compounds exhibited the lowest inhibitory activities due to the lack of a hydrogen‐bonding interaction with the hinge residue L244 (Figure 4). For example, compound 237069 differs from compound 69187 in Class 2 due to the presence of an oxygen atom, which hinders the hydrogen‐bonding interaction with residue L244. As a result, compound 237069 exhibits lower potency than compound 69187. Similarly, compound 320613 differs from compound 322087 by the presence of a formamide group, which disrupts the key hydrogen‐bonding interaction, leading to reduced inhibitory activity. In summary, the interaction analysis revealed the interactions critical for inhibitory potency, including hydrogen bonds with residues E292, E169, and L244, as well as hydrophobic interactions with residue V324.

### 2.5 Cytotoxic and Anti‐Proliferative Effects of Compounds

We selected compounds 150441, 164017, 164016, and 152731 to evaluate their cytotoxicity and anti‐proliferation effect on pancreatic cancer cell lines due to their enzyme inhibitory activities. In addition, compound 322087 was included for testing due to its high IC_50_ value. We conducted experiments on two pancreatic cancer cell lines, Mia PaCa‐2 and Panc‐1, both of which harbor common cancer‐related gene mutations, including *KRAS*, *TP53*, and *CDKN2A*.^[^ ^20^ ^]^ These mutations promote cancer cell proliferation, anti‐apoptosis, and drug resistance. Furthermore, Panc‐1 cells exhibit a more aggressive malignant phenotype compared to Mia PaCa‐2, characterized by the loss of E‐cadherin expression and elevated levels of CDK56 and CD24.^[^ ^21^, ^22^ ^]^

**Figure 5:**
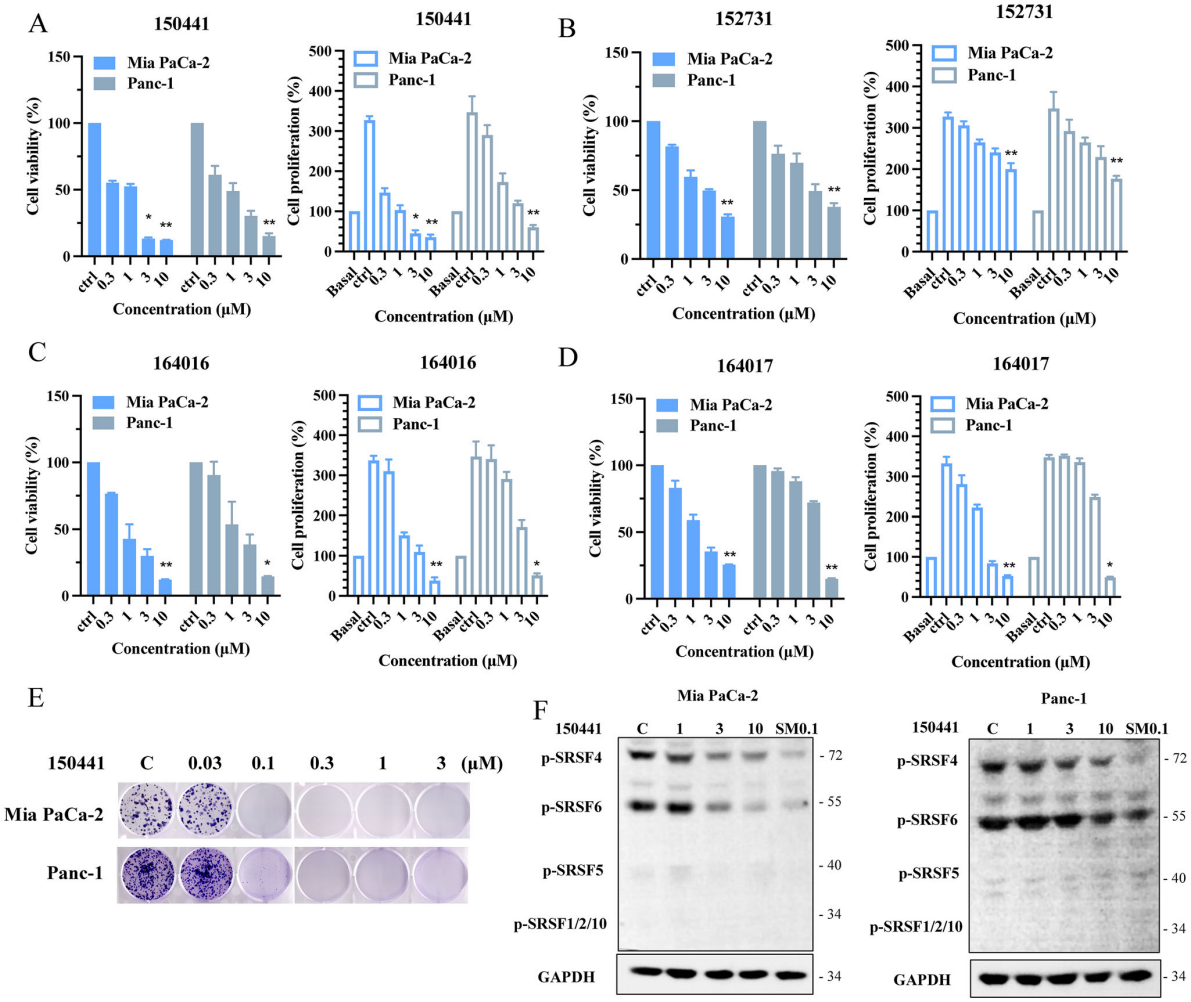
The anticancer effect of CLK4 inhibitors. A–D) Pancreatic cancer cells were treated with compounds at 0.3, 1, 3, and 10 µm for 72 hours. Cell viabilities were determined by 3‐(4,5‐Dimethylthiazol‐2‐yl)‐2,5‐diphenyltetrazolium bromide (MTT) assay, and antiproliferation effects were measured by sulforhodamine B (SRB) assay. E) Colony formation analysis. Cells were incubated with compound 150441 at 0.03, 0.1, 0.3, 1, and 3 µm for 12 days. F) Cells were treated with 150441 at 1, 3, and 10 µm or SM08502 (SM) at 0.1 µm for 2 hours, and the phosphorylation of SR protein was detected by western blotting. SM08502 as a reference compound for SR protein activity inhibition. These results were repeated in at least three independent experiments. ^*^ *p* < 0.05, ^**^ *p* < 0.01 compared to the control (ctrl., untreated) group.

The results indicated that four of these compounds inhibited cell viability and proliferation in both cell lines (**Figure** **5**A–D). Among the tested compounds, 150441 demonstrated the highest potency against cancer cell viability. It significantly reduced cancer cell viability by over 50% at a concentration of 1 µm in both Mia PaCa‐2 and Panc‐1 cell lines (Figure 5A), with IC_50_ values of 0.61 and 0.92 µm, respectively (**Table** **3**). In addition, the compound showed strong anti‐proliferative effects at 0.3 µm in Mia PaCa‐2 cells and at 1 µm in Panc‐1 cells (Figure 5A), with 50% growth inhibitory concentration (GI_50_) values of 0.18 and 0.67 µm, respectively (Table 3). Furthermore, a colony formation assay to evaluate cancer cell proliferation in a 2D culture system showed a concentration‐dependent reduction in both pancreatic cancer cell lines, with the most effective concentrations being 0.1 and 0.3 µm (Figure 5E; Figure S5A, Supporting Information). In addition, compound 150441, demonstrating the strongest enzymatic inhibitory activity with an IC_50_ value of 21.4 nm, exhibited superior potency, achieving over 80% cytotoxicity and more than 95% anti‐proliferative effects at 10 µm in both pancreatic cancer cell lines. In comparison, compound 322087, with lower enzyme potency, showed 40% cytotoxicity in Mia PaCa‐2 and 10% in Panc‐1 at 10 µm, along with 80% antiproliferative activity in Mia PaCa‐2 and 30% in Panc‐1. These findings suggest that compound 150441 has the potential as a novel anticancer drug for pancreatic cancer.

**Table 3:** Cell viability and anti‐proliferative effects of CLK4 inhibitors.

Compound name	Mia PaCa‐2		Panc‐1	
	IC_50_ (Mean ± SD, µm)	GI_50_ (Mean ± SD, µm)	IC_50_ (Mean ± SD, µm)	GI_50_ (Mean ± SD, µm)
150441	0.61 ± 0.1	0.18 ± 0.1	0.92 ± 0.1	0.67 ± 0.1
152731	2.52 ± 0.2	7.67 ± 0.3	3.57 ± 0.4	3.10 ± 0.7
164016	1.02 ± 0.1	0.64 ± 0.1	1.66 ± 0.6	1.27 ± 0.1
164017	1.71 ± 0.1	1.21 ± 0.1	4.09 ± 0.2	1.60 ± 0.1
322087	>10	5.43 ± 0.2	>10	>10

### 2.6 Inhibitory Effect of Compound 150441 on Splicing Regulatory Proteins

CLK4 regulates mRNA splicing by phosphorylating SR proteins, and dysregulation of this process contributes to cancer progression. Inhibiting CLK4 can modulate mRNA splicing and potentially inhibit cancer growth. Therefore, we investigated the effect of compound 150441 on the phosphorylation levels of SR proteins. The compound was tested on two pancreatic cancer cell lines, Mia PaCa‐2 and Panc‐1, at concentrations of 1, 3, and 10 µm for 2 hours. The experimental results showed that compound 150441 significantly inhibited the phosphorylation of SRSF4 and SRSF6 in both cell lines at a concentration of 10 µm (Figure 5F; Figure S5B, Supporting Information). These findings indicate that compound 150441 could affect SR protein activity and subsequently influence the RNA splicing process.

### 2.7 Regulation of Cell Cycle and Induction of Apoptosis By Compound 150 441 Targeting CLK4

**Figure 6:**
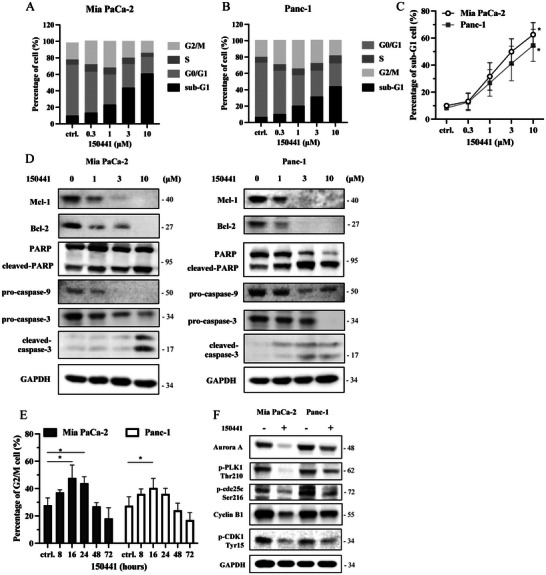
Cancer cell apoptosis and G2/M arrest while 150441 treatment. Flow cytometry analysis was used to assess the distribution of pancreatic cancer cell lines Mia Paca‐2 and Panc‐1 in various cell cycle phases (sub‐G1, G0/G1, S, and G2/M). A,B) After treatment with indicated concentrations of compound 150441 (0.3, 1, 3, and 10 µm) for 72 hours and C) the proportion of cells in the sub‐G1 phase. D) The expression of apoptotic proteins, Mcl‐1, Bcl‐2, PARP, caspase 9, and caspase 3, detected by western blotting. E) Cells were treated with 3 µm compound 150441 for 8, 16, 24, 48, and 72 hours, and the proportion of cells in the G2/M phase. F) The expression levels of G2/M regulatory proteins were assessed following treatment with 10 µm of 150441 for 16 hours. These results were repeated in at least three independent experiments. ^*^ *p* < 0.05 compared to the control (ctrl., untreated) group.

To validate the anticancer properties, we investigated the effect of compound 150441 on the cell cycle distribution of Mia PaCa‐2 and Panc‐1 cell lines. These cells were exposed to 150441 at concentrations of 0.3, 1, 3, and 10 µm for 72 hours. The experimental results revealed a significant concentration‐dependent increase in the proportion of cells in the sub‐G1 phase for both cell lines (**Figure** **6**A–C). Additionally, treatment with 150441 at concentrations of 3 and 10 µm led to a substantial downregulation of the anti‐apoptotic proteins Mcl‐1 and Bcl‐2, along with the activation of the caspase‐dependent apoptotic pathway, as evidenced by an upregulation in cleaved‐caspase levels (Figure 6D).

Additionally, we found that treatment with 150441 at various time points induced G2/M arrest, with the effect being most pronounced at 16 hours (Figure 6E). Previous research has shown that Aurora A enhances PLK1 phosphorylation, which subsequently activates CDC25C through phosphorylation at Ser216. This activation promotes the formation of a checkpoint complex between cyclin B1 and CDK1, thus regulating the transition from the G2 to the M phase.^[^ ^23^, ^24^ ^]^ Our current findings indicated that 150441 significantly suppressed the expression of Aurora A and cyclin B1, while also reducing the phosphorylation of PLK1, CDC25C, and CDK1 (Figure 6F). These results further confirmed that 150441 induced G2/M arrest in cancer cells. Overall, the current results showed that 150441 promoted G2/M arrest and apoptosis in cancer cells by downregulating key regulatory proteins and activating the caspase pathway.

### 2.8 Selectivity Profile of Compound 150441

**Figure 7:**
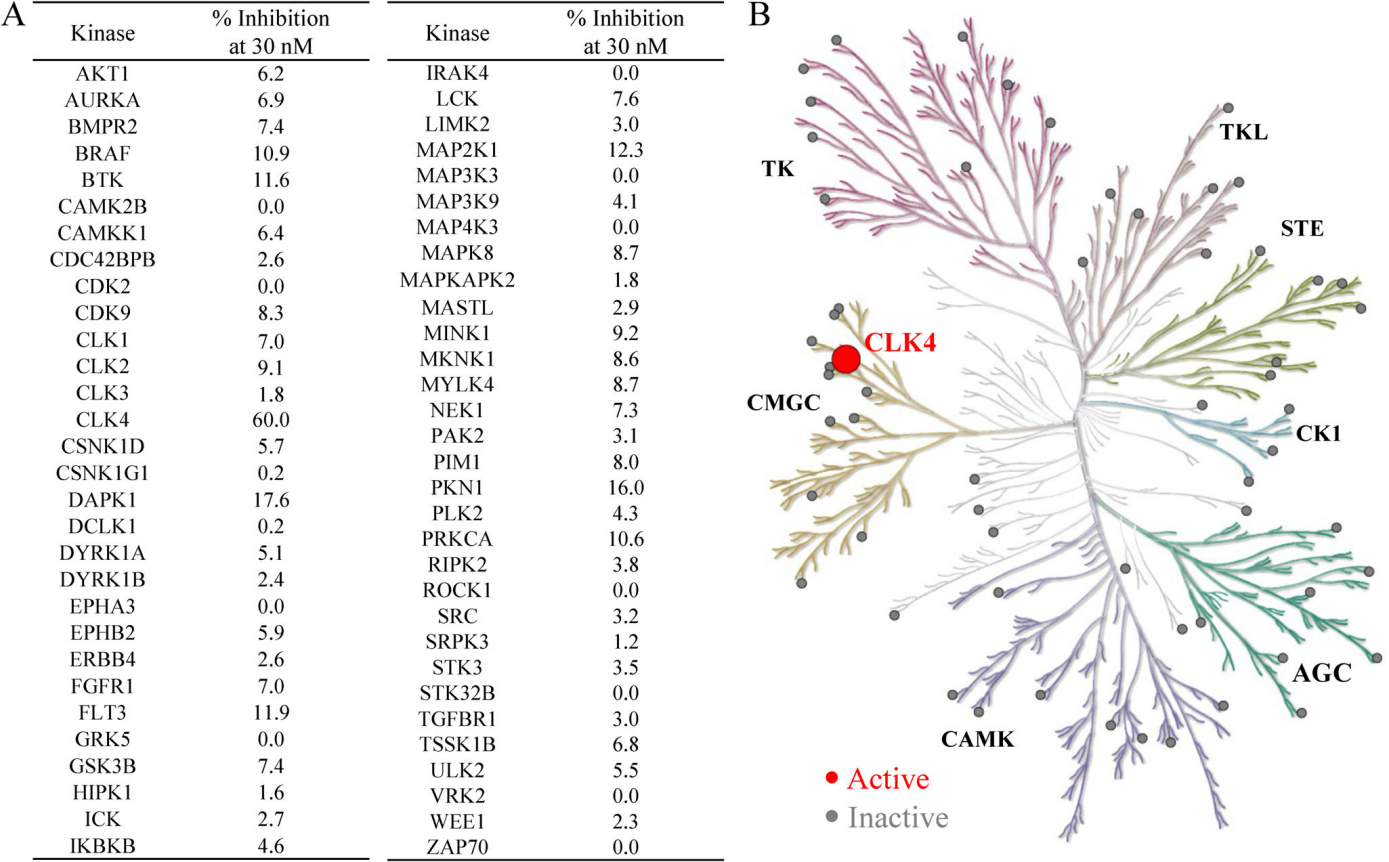
Selectivity profile of compound 150441. A) Inhibitory activity of 150441 on a panel of 60 kinases. B) A kinome tree composed of seven families shows the kinase inhibitory activity of the compound. The compound was tested at a concentration of 30 nm. Among all tested kinases, only the inhibition percentage for CLK4 exceeded 50%, indicated by a red dot.

Selectivity is essential for kinase inhibitors to minimize off‐target effects and reduce adverse reactions. Selective inhibitors also enable researchers to better understand the specific functions of targets like CLK4 in physiological and pathological pathways. Therefore, we further evaluated the selectivity of compound 150441 using kinase profiling analysis. The compound was tested at a concentration of 30 nm against a panel of 60 kinases from various families using Thermo Fisher SelectScreen kinase profiling services (**Figure** **7**). The results showed that compound 150441 specifically targeted CLK4 with an inhibition rate of 60%, suggesting that the compound is a selective inhibitor of CLK4. In addition, CLKs comprise four isoforms: CLK1, CLK2, CLK3, and CLK4. Among the isoforms, compound 150 441 exhibited weaker inhibitory activity against CLK1, CLK2, and CLK3, with inhibition percentages of 7.0%, 9.1%, and 1.8%, respectively (Table S2, Supporting Information). The evidence suggests that compound 150441 is a highly selective CLK4 inhibitor.

### 2.9 RNA‐Seq Analysis of the Effect of Compound 150441

**Figure 8:**
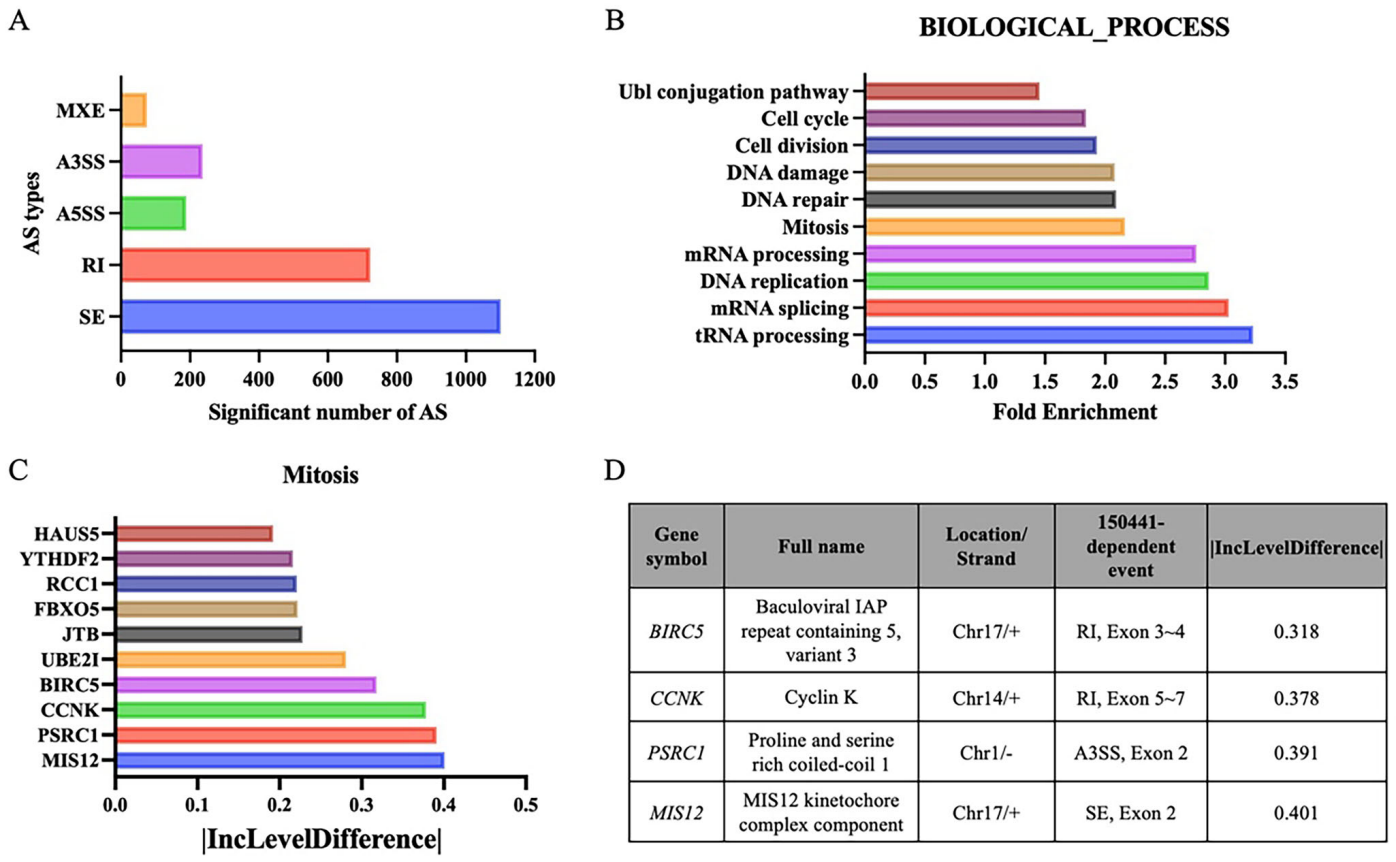
Compound 150441‐induced alternative splicing events in Mia PaCa‐2 cells. Cells were treated with 3 µm compound 150441 for 6 hours and then subjected to RNA‐seq analysis. A) Distribution of splicing event. The number of splicing events caused by compound 150441. B) Gene enrichment analysis. Pathway enrichment of genes affected by 150441‐induced splicing events, analyzed using DAVID gene functional classification. The top ten most enriched biological processes are ranked by fold enrichment. C) Top ten genes in mitosis ranked by |IncLevelDifference|. The genes most affected by 150441 in the mitosis pathway based on splicing inclusion level differences. D) List of genes with |IncLevelDifference| > 0.3 in mitosis.

To investigate the AS events induced by compound 150441, the Mia PaCa‐2 pancreatic cancer cell line was treated with compound 150441 for 6 h, followed by an RNA‐seq analysis. Differential splicing events were identified using replicated multivariate analysis of transcript splicing (rMATS), which categorized five major splicing types: skipped exon (SE), alternative 5′ splice site (A5SS), alternative 3′ splice site (A3SS), mutually exclusive exons (MXE), and retained intron (RI). The results showed that SE was the most frequent splicing event, affecting 1101 genes, followed by RI (723 genes), A3SS (236 genes), A5SS (189 genes), and MXE (75 genes) (**Figure** **8**A). The global increase in AS events caused by compound 150441 suggested the production of numerous aberrant splicing products, likely disrupting essential cellular functions required for cancer cell survival.

To explore the biological processes potentially impacted by compound 150441‐induced splicing changes, we analyzed all detected splicing events using DAVID. Enrichment analysis of the affected genes was performed using the UP_KW_BIOLOGICAL_PROCESS category, and the top 10 significantly enriched pathways, ranked by fold enrichment, are listed in Figure 8B. These results revealed that RNA processing, cell cycle regulation, cell division, and DNA damage response were major biological processes affected by compound 150441 treatment. Interestingly, gene enrichment analysis revealed that the mitosis pathway was associated with compound 150441‐induced splicing events, which is consistent with our in vitro observations of an increased G2/M population after compound 150 441 treatment (Figure 6). To identify key genes involved in the mitotic pathway that were most affected by compound 150441‐induced splicing changes, we used |IncLevelDifference| (the absolute value of inclusion level difference), a measure of the splicing inclusion level difference between conditions. The top 10 genes, ranked by |IncLevelDifference|, are shown in Figure 8C. Among these, *MIS12*, *PSRC1*, *CCNK*, and *BIRC5* were identified as highly affected by 150441, with |IncLevelDifference| values >0.3. The splicing events of these genes are shown in Figure 8D and further visualized using sashimi plots (Figure S6, Supporting Information). These results suggest that compound 150441 significantly altered the splicing of key mitotic regulators, potentially contributing to its effect on cell cycle arrest.

In summary, our RNA‐seq analysis demonstrated that the treatment with compound 150441 led to widespread AS events in Mia PaCa‐2 cells, with several genes implicated in mitotic regulation. These findings provide mechanistic insight into the anticancer effects of compound 150441, particularly through its modulation of splicing in mitotic regulators.

## 3 Discussion

Aberrant AS in pancreatic cancer promotes tumor growth, metastasis, and drug resistance. In this study, we identified a novel therapeutic inhibitor targeting the critical splicing regulatory kinase CLK4 for treating pancreatic cancer. The inhibitor was discovered through an optimized pharmacological model that incorporated pharmacological interactions to enhance the hit rate. It exhibited high selectivity for CLK4, suggesting it may result in fewer side effects. The inhibitor effectively suppressed the activity of SR proteins, SRSF4 and SRSF6, disrupting the RNA splicing process and inhibiting the growth and proliferation of the pancreatic cancer cells. Moreover, the inhibitor promoted cancer cell apoptosis by inducing G2/M cell cycle arrest. Our study elucidates the application of the SBVS approach in developing novel CLK4 inhibitors and provides a therapeutic strategy for targeting RNA splicing in pancreatic cancer treatment.

CLK4 serves as a key regulatory kinase in the AS processes through the activation of SRSF proteins.^[^ ^25^, ^26^ ^]^ CLK4 offers some therapeutic advantages. First, CLK4 inhibition allows for selective modulation of gene expression, enabling precise regulation of oncogenes and tumor suppressor genes in pancreatic cancer. Second, inhibiting CLK4 can address therapy resistance by disrupting splicing‐driven mechanisms, such as *WT1* and *dCK* splicing abnormalities, which are associated with chemotherapy resistance in leukemia.^[^ ^27^ ^]^ Third, CLK4 inhibitors may exhibit synergistic potential when combined with other treatments, such as chemotherapy or immunotherapy, enhancing therapeutic efficacy through complementary mechanisms. Finally, CLK4 inhibition has broader applicability, as splicing abnormalities have been identified in other cancers, including MET gene splicing in lung cancer and exon 7 splicing defects in androgen receptor genes in prostate cancer.^[^ ^28^, ^29^ ^]^ Targeting CLK4 presents versatile and impactful opportunities for advancing cancer therapies, as it plays a pivotal role in RNA splicing across various cancer types.

To investigate the novel interactions generated by compound 150441, we compared its interactions with those of other CLK4 inhibitors, including the 30 known inhibitors, SM08502, and ML315.^[^ ^16^, ^30^ ^]^ The interaction profile revealed that these CLK4 inhibitors mainly interact with residues in the hinge and interior sites. The key differences for compound 150441 are its hydrogen bond with residue E292 in the exterior site and its hydrophobic interactions with residue V225 in the interior site (Figure S7, Supporting Information). Neither SM08502 nor ML315 exhibits these interactions, and only two and seven of the 30 known inhibitors form interactions with E292 and V225, respectively. Notably, SM08502 also interacts with exterior site residues D288 and D325 through its functional groups, piperazine and amide group. In contrast, ML315 lacks the moieties required for interaction with the exterior site. The interaction comparison suggests that compound 150441 forms the two unique interactions, and its distinctive structural feature makes 150441 a promising starting point for developing potent and selective CLK4 inhibitors. In addition, the key interactions identified through the interaction and SAR analyses are summarized in Table S3 (Supporting Information). These consist of ten pharmacological interactions from active compound analysis, including hydrogen bonds with residues K191, E242, and L244, and hydrophobic interactions with residues L167, V175, A189, K191, L244, L295, and V324. Two additional key interactions were identified from the SAR analysis, namely hydrogen bonds with residues E169 and E292. Together, these interactions provide valuable insights for the identification and design of CLK4 inhibitors.

Selectivity of kinase inhibitors is a crucial issue in drug discovery. However, most CLK4 inhibitors exhibit off‐target inhibitory actions, leading to unexpected side effects. For example, SM08502 (Cirtuvivint), the first small‐molecule CLK inhibitor to enter clinical trials, inhibits multiple kinases, such as MAP4K4, MINK1, and LRRK2.^[^ ^31^ ^]^ Research suggests that LRRK2 inhibition could lead to the development of intestinal‐immune diseases.^[^ ^32^ ^]^ Previous studies have shown that targeting Msn kinases (MINK1 and MAP4K4) can promote regenerative proliferation.^[^ ^33^ ^]^ ML315, another potent CLK4 inhibitor with an IC_50_ value of 68 nm, inhibits CLK1 (68 nm) and CLK2 (231 nm). ML315 also inhibits kinases in other families, such as CSNK1E, MAP3K1, PKNB, and PRKCE, which may lead to potential off‐target effects. In comparison, the selectivity profiling results of compound 150441 showed that it is a selective CLK4 inhibitor without inhibiting CLK1, CLK2, and CLK3. The compound also did not exhibit inhibitory activity against members of other kinase families. These results suggest that compound 150441 is less likely to cause adverse effects due to its high selectivity.

CLK4 facilitates the splicing process by regulating the phosphorylation of SRSF proteins.^[^ ^25^, ^26^ ^]^ Our findings demonstrate that the CLK4 inhibitor, compound 150441, significantly reduced the phosphorylation of SRSF4 and SRSF6 (Figure 5F). SRSF proteins consist of 12 isoforms, SRSF1 to SRSF12, based on their structural differences.^[^ ^34^ ^]^ Each isoform possesses unique arginine/serine‐rich (RS) domain sequences and structural configurations, which determine its affinity for CLK4 and its sensitivity to CLK4 inhibition.^[^ ^35^, ^36^ ^]^ Differences in the effects of CLK4 inhibitors on SRSF isoforms may arise from variations in their chemical structure and selectivity. For example, SM08502, which inhibits DYRK and CLK kinases, primarily targets SRSF5 and SRSF6,^[^ ^16^ ^]^ while ML167 mainly inhibits SRSF1.^[^ ^15^ ^]^ In addition, SRSF isoforms may regulate different splicing targets. For instance, SRSF1 regulates the splicing of genes, such as cyclin D1, MNK2, and RON1,^[^ ^37^, ^38^, ^39^ ^]^ while SRSF2, SRSF4, SRSF5, and SRSF6 are involved in the splicing of apoptosis‐related genes.^[^ ^40^, ^41^, ^42^, ^43^ ^]^ SRSF6 predominantly controls the splicing of EMT‐related genes.^[^ ^44^ ^]^ Therefore, the variations in CLK4 and SRSF protein expression levels in cancer cells, along with the differences in the chemical structure and selectivity of the compounds, lead to diverse inhibitory responses among SRSFs. This complexity underscores the importance of selecting the most suitable inhibitor to effectively target specific SRSF proteins in therapeutic applications.

Compound 150441 induced G2/M arrest in cancer cells, ultimately leading to apoptosis. The results indicated that compound 150441 effectively inhibited the expression of Aurora A and cyclin B1 and the activation of PLK1, CDK1, and CDC25C, causing cancer cell arrest in the G2 phase (Figure 6). However, CLK4 does not directly participate in regulating the cell cycle. Given the role of CLK4 in RNA splicing, CLK4 inhibition affects the splicing of cyclin B1, thereby reducing the production of the cyclin B1 protein. This reduction prevents the formation of the CDK1‐cyclin B1 complex necessary for regulating the G2/M phase.^[^ ^45^ ^]^ Moreover, previous studies have shown that the alterations in the splicing of CDC25C can disturb the proper activation of CDK1, preventing cells from entering mitosis and thereby maintaining them in the G2 phase.^[^ ^46^, ^47^ ^]^ Consequently, the inhibition of CLK4 can significantly impact the regulation of the G2/M phase by modifying the AS of key genes involved in cell cycle progression. By influencing cyclin B1, CDK1, and other critical regulators, CLK4 inhibition disrupts the proper transition from the G2 to M phase, resulting in cell cycle arrest. This mechanism presents potential therapeutic applications, particularly in cancer treatment, by sensitizing cells to DNA damage and enhancing the efficacy of treatments that depend on inducing G2/M arrest.

RNA‐seq results revealed that compound 150441 induced extensive splicing alterations in various genes. Among these, *MIS12*, *PSRC1*, *CCNK*, and *BIRC5* exhibited the most significant splicing changes within the mitosis pathway (**Figure** **8**). These splicing events may alter wild‐type protein expression and produce isoforms that impair native protein functions. *MIS12* encodes the Mis12 protein, which plays a critical role in kinetochore assembly during mitosis.^[^ ^48^ ^]^

The splicing changes observed in *MIS12* induced by compound 150441 may impair the production of functional Mis12 protein, compromising its ability to bind centromere‐specific proteins. This disruption could lead to chromosome misalignment and delayed mitotic progression, and reduced Mis12 levels are known to impair proper chromosome segregation.^[^ ^49^ ^]^ Similarly, *PSRC1* encodes DDA3, a microtubule‐associated protein that regulates mitotic spindle dynamics by interacting with Kif2a.^[^ ^50^ ^]^ Splicing alterations in *PSRC1* likely alter DDA3 function, which has been linked to unaligned chromosomes during mitosis.^[^ ^51^ ^]^ In addition, *CCNK* encodes cyclin K, a regulator of chromosome segregation that interacts with Aurora kinase to ensure accurate mitotic progression.^[^ ^52^ ^]^ Splicing changes in *CCNK* induced by compound 150441 may impair cyclin K function, further disrupting mitotic control. Collectively, the RNA‐seq analysis suggested that compound 150441 likely induced mitotic arrest through AS events in *MIS12*, *PSRC1*, and *CCNK*. These findings provide a potential explanation for its anticancer mechanism via G2/M cell cycle arrest, as observed in Figure 6. Interestingly, CLK inhibitors, such as cpd‐2 and TG003, have been reported to impact the cell cycle.^[^ ^53^, ^54^ ^]^ TG003 promoted the splicing of *CENPE* into its long isoform, leading to mitotic defects,^[^ ^54^ ^]^ while compound 150441 induced mitotic defects by AS in *MIS12*, *PSRC1*, *AURKB*, and several cyclin regulators (Supporting information).

Another gene affected by compound 150441 through splicing alterations is *BIRC5*, which encodes the antiapoptotic protein Survivin. As an inhibitor of apoptosis protein, Survivin plays a critical role in cellular stress responses during prolonged mitotic arrest.^[^ ^55^, ^56^ ^]^ Survivin has been shown to activate the DNA damage repair machinery^[^ ^57^ ^]^ and is associated with chemoresistance in PDAC.^[^ ^58^ ^]^ Compound 150441 induced intron retention in *BIRC5*, which may impact the production of functional Survivin (Figure S6, Supporting Information). Notably, distinct splice variants of Survivin exhibit distinct functions, with some isoforms acting as pro‐apoptotic proteins.^[^ ^59^ ^]^ Therefore, the splicing alterations induced by compound 150441 may reduce the anti‐apoptotic effect of Survivin. In conclusion, the potential mechanisms of compound 150441 include disrupting mitotic progression by altering the splicing of key mitotic regulators, such as *MIS12*, *PSRC1*, and *CCNK*, and impairing survival pathways through splicing alterations in *BIRC5*, which encodes Survivin. These multiple effects are especially important because PDAC patients show elevated Survivin expression, which is strongly associated with increased drug resistance.^[^ ^60^ ^]^

## 4 Conclusion

CLK4 represents a promising therapeutic target for the treatment of pancreatic cancer. In this study, we developed an optimized pharmacological model incorporating pharmacological interactions and successfully identified novel CLK4 inhibitors. Among the inhibitors, compound 150441 demonstrated the most potent inhibitory activity with an IC_50_ value of 21.4 nm. The SAR analysis revealed the key functional groups and interactions contributing to its efficacy. Kinase profiling further confirmed the selectivity of compound 150441 for CLK4. Moreover, this compound inhibited the growth and survival of pancreatic cancer cells by inducing G2/M phase cell cycle arrest. The treatment with compound 150441 also inhibited the phosphorylation of SRSFs. RNA‐seq analysis further demonstrated that compound 150441 induced widespread alternative splicing changes, particularly affecting genes involved in RNA processing, DNA replication, DNA damage, and cell cycle regulation. These findings suggest that compound 150441 is a promising starting point for further optimization as a new pancreatic cancer treatment.

## 5 Experimental Section

### Molecular Docking and Identification of Pharmacological Interactions

Known CLK4 inhibitors with a IC_50_ value of ≤1 µm were obtained from the ChEMBL compound database. These compounds were clustered using Pipeline Pilot,^[^ ^61^ ^]^ resulting in the selection of 60 diverse compounds. The protein structure of CLK4 (PDB ID: 6FYV) was obtained from the RCSB Protein Data Bank.^[^ ^62^ ^]^ Structures of the protein and the compounds were prepared using the Maestro software suite.^[^ ^63^ ^]^ The co‐crystal ligand was used as the centroid for the docking grid. Compounds were docked into the CLK4 binding site using Glide,^[^ ^64^ ^]^ and the top compounds were selected for a further interaction analysis using Pipeline Pilot. In this study, pharmacological interactions were defined as hydrogen‐bonding or hydrophobic interactions that appeared in ≥50% of the docked CLK4 inhibitors.

### Virtual Screening for Potential CLK4 Inhibitors

The NCI compound library, containing ≈280 000 compounds, was selected for screening. Pipeline Pilot was used to filter out compounds with poor drug properties. The initial screening involved a high‐throughput screening (HTS) filter to remove poor candidates, including molecules with non‐organic atom types and reactive structures. Compounds that violated the “Lipinski and Verber Rules” or contained Pan Assay Interference Structures (PAINS) were also removed. In addition, compounds with a calculated QED score of ≤0.25 were removed. The remaining compounds were docked into the CLK4 binding site. The final pharmacological interaction scores were then calculated and ranked. Finally, 15 compounds were selected for evaluation based on their availability, inspection, and structure similarity.

### Kinase Assay

Enzyme‐based assays were carried out by Thermo Fisher Scientific (Waltham, MA, USA). Thermo Fisher Scientific utilizes LanthaScreen Eu Kinase Binding Assay Technology (www.thermofisher.com/lanthascreen), which is based on fluorescence resonance energy transfer (FRET), to conduct the kinase activity assays. Fluorescent receptor dyes are conjugated to the kinase, and an Eu‐labeled anti‐tag antibody is added to detect the phosphorylated fluorescein‐labeled substrate. The fluorescence‐labeled kinase, antibody, and test compounds are combined in an appropriate buffer solution. After incubation, the mixture is placed in a fluorescent plate reader capable of detecting FRET signals. FRET efficiency is calculated by comparing the ratio of the acceptor emission to the donor emission. The selected compounds were evaluated at specified concentrations, and IC_50_ values were determined using GraphPad Prism software (La Jolla, CA, USA). Each enzymatic activity assay was performed in duplicate, following the quality control guidelines of Thermo Fisher Scientific. Kinase profiling was carried out using the SelectScreen kinase profiling service provided by Thermo Fisher Scientific, with supplementary assays such as Adapta and Z'LYTE included when available for the respective kinases. The detailed protocols are available at the following links: www.thermofisher.com/adapta and https://www.thermofisher.com/z‐lyte.

### Cell Culture

Pancreatic cancer cell lines, Mia PaCa‐2 and Panc‐1, were obtained from the Bioresource Collection and Research Center (BCRC, Hsinchu, Taiwan). These cells were cultured in Dulbecco's modified Eagle medium (DMEM) medium supplemented with 10% (v/v) fetal bovine serum (FBS), 100 units/mL of penicillin, and 100 µg mL^−1^ of streptomycin. Cultures were maintained at 37 °C in a humidified incubator with 5% CO_2_.

### Cell Viability Analysis

Cells were seeded in 96‐well plates at specified densities: 3 × 10^3^ cells per well for Mia PaCa‐2 cells and 5 × 10^3^ cells per well for Panc‐1 cells. After cells had become adhered, they were treated with concentrations of 0.3, 1, 3, and 10 µm of the compounds for 72 hours. The MTT reagent (0.5 mg mL^−1^ in PBS) was then added to the medium at a 1:10 ratio, and the plates were incubated for 1 hour. Following incubation, 100 µL of DMSO was added to each well to dissolve the MTT metabolic products.^[^ ^65^ ^]^ The absorbance was measured at 550 nm (Synergy HTX ELISA reader, Bioteck, CA, USA), and IC_50_ values were calculated based on cell viability.

### Cell Proliferation Analysis

Mia PaCa‐2 cells were seeded at 3 × 10^3^ cells per well, while Panc‐1 cells were seeded at 5 × 10^3^ cells per well, both in 96‐well plates. After allowing the cells to adhere, they were exposed to concentrations of 0.3, 1, 3, and 10 µm of the compounds for 72 hours. Following treatment, cells were fixed with 10% trichloroacetic acid (TCA) for 15 min and rinsed with distilled water. Subsequently, 100 µL of 0.4% SRB dye was applied for staining, and cells were then washed with 1% acetic acid.^[^ ^66^ ^]^ Finally, 100 µL of 10 mm Tris‐base was added to each well, and the absorbance was read at 515 nm to calculate GI_50_ values based on cell proliferation (Synergy HTX ELISA reader, Bioteck, CA, USA).

### Western Blot Analysis

Cells were treated with the compound at the indicated concentrations and incubation times. After treatment, cell lysates were extracted using RIPA buffer containing phosphatase inhibitors (2 mm Na_3_VO_4_, 1 mm NaF, and 20 mm NaP_2_O_4_) and an EDTA‐free protease inhibitor. The lysates were centrifuged at 14 000 rpm for 15 min at 4 °C, and total protein content was measured using a BCA protein assay kit. For sample preparation, lysates were mixed with 5X sample buffer (312.5 mm Tris, pH 6.8, 10% sodium dodecylsulfate (SDS), 50% glycerol, 0.05% bromophenol blue, and 10% 2‐mercaptoethanol) and incubated at 95 °C for 10 min.

For the western blot analysis, equal amounts of protein were separated by SDS‐PAGE and transferred onto PVDF membranes. The membranes were then blocked with 5% nonfat milk in TBST buffer for 1 hour at room temperature. Target proteins were detected by incubating the membranes with primary antibodies in TBST overnight at 4 °C. The following day, the membranes were washed with TBST and incubated with HRP‐conjugated secondary antibodies for 1 hour at room temperature. Primary antibodies phospho‐SR proteins (MABE50) and GAPDH (MAB374) were purchased from Millipore (Bedford, MA, USA). The Mcl‐1 antibody (SC‐819) was purchased from Santa Cruz (Santa Cruz, CA, USA), and Cyclin B (BD554178) and PLK1 (Thr210) (BD558400) were obtained from BD Biosciences (Qume Drv, USA). The antibodies against Bcl‐2 (2876S), PARP, caspase 9 (#9502), caspase 3 (#9662), cleaved caspase 3 (#9661), Aurora A (#4718), CDC25C (Ser216) (#9528), and CDK1 (Tyr15) (#9111) were purchased from Cell Signalling Technology (MA, USA). The secondary HRP‐conjugated anti‐rabbit IgG (111‐035‐003) and anti‐mouse IgG (115‐035‐003) were obtained from Jackson ImmunoResearch (PA, USA). Protein expression was visualized using an enhanced chemiluminescence detection kit and detected using an eBLOT machine (Shanghai, China). Relative protein expression levels were quantified with Image J software (National Institutes of Health (NIH), Bethesda, MD, USA).

### Cell Cycle Analysis

The cell cycle was analyzed using flow cytometry. Cells (3 × 10⁵ cells/well) were plated in 6‐cm dishes with 3 mL of culture medium and exposed to various concentrations of the compound for specified time intervals. Following treatment, cells were harvested, washed with cold PBS, and fixed in 70% ice‐cold ethanol at −20 °C for 30 min. After fixation, cells were centrifuged to remove ethanol and subsequently stained with 0.5 mL of propidium iodide (PI) staining buffer, which contained 80 µg mL^−1^ PI, 100 µg mL^−1^ RNase A, and 1% Triton X‐100 in PBS, for 20 min. The cell cycle distribution was then determined using a BD Accuri™ Flow Cytometer and its accompanying software (Becton Dickinson, Mountain View, CA, USA).

### RNA Sequence Analysis

Mia PaCa‐2 cells were exposed to 3 µm of the compound for 6 hours, after which total RNA was isolated according to the Direct‐zol RNA Kit protocol (Zymo Research, CA, USA). The RNA purity and concentration were measured using SimploNamoE‐Biochrom Spectrophotometers (Biochrom, MA, USA). Sequencing libraries were generated from 1 µg of total RNA using the KAPA mRNA HyperPrep Kit (KAPA Biosystems, Roche, Switzerland). The quality of the libraries was evaluated using the Qubit 2.0 Fluorometer (Thermo Scientific, MA USA) and the Agilent Bioanalyzer 2100 system. Finally, sequencing was performed on the Illumina NovaSeq 6000 platform. Differential AS events were analyzed with rMATS.^[^ ^67^ ^]^ Splicing events were identified using splice junction exon counts (JCECs), with significant events defined as those with a false discovery rate (FDR) <0.05 and related reads ≥10 in either splicing form. For pathway enrichment analysis, DAVID (The Database for Annotation, Visualization, and Integrated Discovery)^[^ ^68^ ^]^ was used to identify biological processes enriched in the genes affected by compound‐induced splicing changes.

### Statistical Analysis

All biological experiments were repeated at least three times to ensure reproducibility and reliability of the results. The data are expressed as the mean ± standard deviation (SD) or as a percentage of the control group, depending on the nature of the data. For statistical analysis, a Student's *t*‐test was used to compare the differences between the treatment and control groups. In some cases, a two‐tailed Student's *t*‐test was applied to determine the significance of differences. A *p*‐value of <0.05 was considered statistically significant. Additionally, a one‐way analysis of variance (ANOVA) was performed to analyze the data. When the ANOVA indicated significant differences between groups, Tukey's post‐hoc test was used to identify which pairs of groups showed statistically significant differences. Parameters with *p* < 0.05 were deemed statistically significant.

## Conflict of Interest

The authors declare no conflict of interest.

## Author Contributions

C.‐L.Y. and Y.‐W.W. contributed equally to this work. C.‐L.Y., Y.‐W.W., S.‐L.P., and K.‐C.H. were responsible for designing the experiments and writing the manuscript. C.‐L.Y., T.E.L., and T.‐Y.S. performed molecular docking and analyzed the results. Y.‐W.W., M.‐C.L., and S.‐C.Y. analyzed the results of kinase profiling. Y.‐W.W., Y.‐H.Y., and H.‐J.T. established the experimental models of SR protein phosphorylation, and regulatory protein expression, and analyzed the data. H.‐J.T. and J.‐H.H. performed the NGS and gene enrichment analysis. S.‐Y.H., M.‐C.Y., and H.‐P.H. c provided resources, and funding, and oversaw the investigation. S.‐L.P. and K.‐C.H. conceived and supervised the project. All authors have read and agreed to the published version of the manuscript.

## Supporting information



Supporting Information

Supplemental Table 1

## Data Availability

The data that support the findings of this study are available from the corresponding author upon reasonable request.
