# Supplementary material for: Identification and Biological Evaluation of a Novel CLK4 Inhibitor Targeting Alternative Splicing in Pancreatic Cancer Using Structure‐Based Virtual Screening
Source: Adv Sci (Weinh). 2025 Mar 24;12(19):2416323. doi: 10.1002/advs.202416323 (PMC12097107; doi:10.1002/advs.202416323)
Supplement: Supplementary file 1 — Supporting Information [file ADVS-12-2416323-s002.docx]

**Table S1. Inhibitory activity of selected compounds.**

**Table S2. The inhibitory activities of compounds on CLK kinase family.**

| **Compound** | **% Inhibition at 1 μM** | | | |
| --- | --- | --- | --- | --- |
|  | **CLK1** | **CLK2** | **CLK3** | **CLK4** |
| 150441 | 75 | 62 | 6 | 98 |
| 164017 | 68 | 58 | 12 | 96 |
| 164016 | 65 | 69 | 7 | 95 |
| 152731 | 86 | 81 | 4 | 98 |

**Table S3. Summary of key interactions.**

| **Type** | **Residues** |
| --- | --- |
| Hydrogen-bonding interactions | E169, K191, E242, L244, E292 |
| Hydrophobic interactions | L167, V175, A189, K191, L244, L295, V324 |

**Figure S1. Comparison of co-crystal and re-docking poses.** CLK4 is shown in wheat. Co-crystal and re-docking poses are shown in green and pink, respectively.

**Figure S2.** **Optimization of pharmacological model by analyzing inactive compounds.** (A) Interaction frequencies of active and inactive compounds. The brown line represents the difference in frequency for each interaction. M, P, and HB denote mixed hydrophobic interactions, Pi-stacking interactions, and hydrogen-bonding interactions, respectively. (B) ROC curve generated using the pharmacological score and the optimized pharmacological scores.

**Figure S3.** **The docking pose of compound 150441 in CLK4.** The CLK4 structure is rendered as a cartoon, and 150441 is rendered as salmon line.

**Figure S4. The inhibitory effect of compounds against CLK4 kinase.**

**Figure S5. The effect of compound 150441 in anticancer and SR protein phosphorylation.** (A) The quantitative results of colony formation assays following treatment with compound 150441. (B) The expression levels of phosphorylated SRSF4 and SRSF6 were detected by western blotting, and the corresponding quantitative results are shown. These results were repeated at least three independent experiments. **p* < 0.05, ****p* < 0.001 compared to the control (ctrl., untreated) group.

**Figure S6. SX Quantitative visualization of alternative splicing events induced by compound 150441.** RNA-Seq read alignments for (A) *BIRC5*, (B) *CCNK*, (C) *PSRC1*, and (D) *MIS12* are shown. Each arc represents a splice junction connecting two exons, with the thickness reflecting the number of supporting RNA-Seq reads. The numerical values adjacent to the arcs indicate the frequency of the splicing events, providing a quantitative assessment of junction usage.

**Figure S7. Comparison of interactions between known inhibitors and compound 150441.** (A) The interaction profile of SM08502, ML315, and compound 150441. The average percentages of interaction frequencies are generated using the 30 known inhibitors. (B) Binding poses of SM08502, ML315, and compound 150441. Exterior, hinge, and interior residues are shown in lime, blue, and pink, respectively.
